# Extracting the Behaviorally Relevant Stimulus: Unique Neural Representation of Farnesol, a Component of the Recruitment Pheromone of *Bombus terrestris*

**DOI:** 10.1371/journal.pone.0137413

**Published:** 2015-09-04

**Authors:** Martin F. Strube-Bloss, Austin Brown, Johannes Spaethe, Thomas Schmitt, Wolfgang Rössler

**Affiliations:** 1 Department of Behavioral Physiology & Sociobiology, Theodor-Boveri-Institute of Bioscience, Biocenter University of Würzburg, Am Hubland, 97074, Würzburg, Germany; 2 Department of Molecular and Cellular Biology, University of Arizona, Life Sciences South Building, 1007 E. Lowell Street, Tucson, AZ, 85721, United States of America; University of Paris 13, FRANCE

## Abstract

To trigger innate behavior, sensory neural networks are pre-tuned to extract biologically relevant stimuli. Many male-female or insect-plant interactions depend on this phenomenon. Especially communication among individuals within social groups depends on innate behaviors. One example is the efficient recruitment of nest mates by successful bumblebee foragers. Returning foragers release a recruitment pheromone in the nest while they perform a ‘dance’ behavior to activate unemployed nest mates. A major component of this pheromone is the sesquiterpenoid farnesol. How farnesol is processed and perceived by the olfactory system, has not yet been identified. It is much likely that processing farnesol involves an innate mechanism for the extraction of relevant information to trigger a fast and reliable behavioral response. To test this hypothesis, we used population response analyses of 100 antennal lobe (AL) neurons recorded in alive bumblebee workers under repeated stimulation with four behaviorally different, but chemically related odorants (geraniol, citronellol, citronellal and farnesol). The analysis identified a unique neural representation of the recruitment pheromone component compared to the other odorants that are predominantly emitted by flowers. The farnesol induced population activity in the AL allowed a reliable separation of farnesol from all other chemically related odor stimuli we tested. We conclude that the farnesol induced population activity may reflect a predetermined representation within the AL-neural network allowing efficient and fast extraction of a behaviorally relevant stimulus. Furthermore, the results show that population response analyses of multiple single AL-units may provide a powerful tool to identify distinct representations of behaviorally relevant odors.

## Introduction

Innate behaviors should be hardwired, meaning that animals respond with a more or less stereotypic behavior when confronted with a specific stimulus or combination of stimuli [1]. In flies, attraction to some food-related odors depends only on the activation of a single type of olfactory receptor [2]. In the same way, pheromones trigger numerous innate behaviors [3]. In insects, sex-pheromone specific pathways have been identified morphologically and physiologically [4–10]. For olfactory systems it is assumed that transduction and neural processing of a pheromone involves spatially segregated pathways: beginning with the level of odorant receptors (OR), to the olfactory sensory neurons (OSN) via specialized glomeruli of the antennal lobe (AL), and further to higher order brain centers, where information is evaluated and finally relayed to appropriate motor control centers that trigger a predetermined behavior. It is still under debate whether such a so called ‘labeled line’ is a fixed structure (hard wired) or if it includes interactions of the sensory neuronal networks at different processing levels. The latter is supported by inhibitory interactions between the pheromonal and the non-pheromonal subsystem of the AL reviewed by Heinbockel and collegues [11]. In the moth *Heliothis virescens* it is shown that plant odorants can interfere with the signaling of a major component of a sexpheromone already at the receptor level [12]. In another moth species (*Agrotis ipsilon*) computational interactions between pheromone and general odors are demonstrated to occurs at the receptor level [13] and at the AL-network level [13,14]. But also computation of different single pheromone components suggest a broad interaction at the AL-level in honeybees reflected in their combinatorial projection neuron (PN) activity [15]. Furthermore, it is still unclear whether spatial segregation at the AL level alone allows separation of pheromone components from other chemically related odors or if a pre-tuned neuronal network at early levels of information processing already contributes to the separation and categorization of the particular valence of a behaviorally relevant stimulus. Evidence for the latter comes from honeybees, where the segregation between pheromone types at the AL-level is passed into the lateral horn where it stays separated [16].

In the present study we introduce a system that allows to investigate stimulus separation of chemically related, but behaviorally distinct odor components at the AL level and characterized their neural representation in the AL-network of the bumblebee, *Bombus terrestris*.

Honeybees and bumblebees are well established model organisms to investigate olfactory learning and memory formation [17–21]. Moreover, extracellular long-term recordings in honeybees have been established to characterize learning-related plasticity in mushroom body output neurons after classical conditioning [22], showing that neurons at this processing level encode the odor reward associations [23]. The experimental as well as the analytical methods we adopted here are therefore adequate to investigate and identify the representation of a behaviorally relevant stimulus out of the activity of a neural population. The social organization of bumblebees includes a variety of olfactory communication. For example, the recruitment pheromone, released by successful foragers inside their nest, induces increased movement speed and searching behavior in unemployed workers after a period of food shortage [24,25]. Using gas chromatography and mass spectrometry of headspace samples from activated and non-active bumblebee colonies, Granero et al. [26] identified three main components, which were only present when successful foragers returned to the nest. One of them was farnesol with the largest relative and absolute increase after colony activation in both the tergal glands and the headspace above the nest [26]. Farnesol is a acyclic sesquiterpene alcohol and was shown to be used by several bee species in the context of pheromone communication (honeybees:[27]; mining bees: [28]; bumblebees:[29,30]).

The first-order olfactory neuropil of the insect brain, the AL, shows analogous organization compared to the vertebrate olfactory bulb (for review cp. [31,32]). The AL’s morphological subunits are glomeruli, receiving input via OSNs from the antenna. Glomerular output is transferred to higher centers by olfactory PNs. OSNs and PNs are connected either via direct synapses or via different types of local interneurons (LN) that shape the spatio-temporal response pattern of the glomeruli [33–35]. Olfactory information of the AL is further transmitted by PNs, mainly via the medial and the lateral antennal lobe tract (m-ALT and l-ALT) to higher order processing centers, such as the mushroom bodies and the lateral horn [36–42]. Using electro- antennogram recordings Fonta and Masson [43] already compared the response of single floral and pheromonal odor components at the OSN-level in bumblebees. Here we move one step further along the olfactory pathway by focusing on odor processing within the AL-network. We investigate whether farnesol, a major component of the foraging pheromone in bumblebees, is processed differently compared to other, chemically related odors involved in the olfactory location of floral resources by analyzing and comparing AL-activation patterns of farnesol with those induced by related terpene-alcohols (geraniol and citronellol) and the terpene-aldehyde citronellal [44–46]. We performed extracellular long-term multi-unit recordings to extract single unit activity of 100 AL-neurons from 25 bumblebees and analyzed their odor induced population activity.

## Materials and Methods

### Animals

No specific permissions were required. Bumblebee (*Bombus terrestris*) colonies were obtained from a commercial breeder (Koppert B. V., Netherlands) and kept in a climate chamber under controlled laboratory conditions (humidity: 50%; temperature: 25°C; light dark cycle: 12h/12h). In the morning before the experiment started, workers were caught and harnessed as described in Sommerlandt et al. [21]. Using eicosane (Sigma-Aldrich, Taufkirchen, Germany) the heads of the animals were glued to the holders and the scapi of both antennae were fixed onto the head capsule before dissecting the animals. A small window (1.5 x1.5 mm) was cut in the cuticle between the compound eyes close to the base of the antennae. Glands and trachea above the antennal lobe (AL) were carefully removed before the electrode was inserted at the dorsal rim of the AL at a depth between 150–300 μm, where the medial (m-) and the lateral (l-) antennal lobe tract (ALT) exit the AL. The brain was then covered with a droplet of two component silicon (1:1 mixture, KWIK-SIL Sarasota, FL, USA) to fix the electrode relative to the brain and to prevent it from drying out.

### Odor stimulation

A 12-channel olfactometer similar to the one described earlier [23,47] was used for stimulation. Each odor channel was equipped with a syringe (5 ml). A constant air stream (25 ml/s) was filtered through water and delivered through a Teflon tube (10 mm in diameter) into which the needles of the syringes were inserted. During odor stimulation the air stream was switched between an empty syringe and a syringe containing the odor to avoid air fluctuations and mechanical stimulation. Odors were diluted in fresh paraffin oil (1:100, Sigma Aldrich GmbH, Germany), which consists of n-alkanes (saturated carbon chains) which do not react with the terpenoids. N-Alkanes are found in bee wax and are part of the cuticular hydrocarbon layer. They have also been suggested to be involved in nest mate recognition [48]. We therefore consider paraffin oil as an olfactory background to which we add the particular odor component. Filter papers (2 cm^2^) were soaked with 6 μl of odor solution and placed in the syringes. During the three-second odor stimulation, only half (2.5 ml) of the syringe air volume was injected into the constant air stream. The olfactometer was controlled via TTL pulses which were generated and synchronized to the recording channels using the trial control software from Neuralynx (Bozeman, MA, USA). We tested four different odor stimuli, geraniol (CAS#: 106-24-1), citronellol (CAS#: 106-22-9), citronellal (CAS#: 106-23-0) and farnesol (CAS#: 4602-84-0) all purchased from (Sigma Aldrich GmbH, Germany). Each stimulus application was repeated ten times in a pseudo-randomized order.

### Data acquisition

We used a 16-channel digital data acquisition system from Neuralynx. The extracellular electrode consisted of three micro wires (polyurethane-coated copper wire, 14 μm in diameter, Electrisola, Escholzmatt, Switzerland). Detailed instructions of electrode building and visualization of the recording position are described in detail elsewhere [49]. A silver wire with a diameter of 25 μm (Nilaco, Tokyo, Japan) was used as reference electrode and inserted into one compound eye. Wires were connected to a head stage preamplifier (HS-16, Neuralynx). Neural activity was measured differentially from all pair-wise combinations of the three wires using the Cheetah data acquisition software (Cheetah 5, Neuralynx) with a sampling rate of 30 kHz and high-pass filtered (>600 Hz).

### Spike sorting

We applied a semi-automatic spike sorting technique (template-matching) provided with the Spike2 software (Cambridge Electronic Design, Cambridge, UK). For details see [23,47,49]. In brief, we calculated the mean activity and standard deviation (SD) of the high-pass filtered channels and set the thresholds for detecting events at ± 3 SDs. Threshold crossing events were used to compute templates of spike waveforms which were subsequently used to assign individual spikes. We used principal component analysis (PCA) of the detected waveforms and inter-spike-interval distribution to control for sufficient unit separation. In total we separated 100 units from 25 bumblebees. Recording electrodes were inserted directly into the AL at the region were the l- and m-ALT exit the AL to project to the MB and the LH. Although the projection neurons of the tracts may dominate the signal at this area, it is still possible that some local interneurons (LN) were recorded. Therefore we use the term AL-neuron (unit) throughout the text to stress the fact that we recorded and sorted single-unit activity from the antennal lobe network.

### Analysis

Data analysis was carried out with MatLab and the FIND open source toolbox ([50]; http://find.bccn.uni-freiburg.de/). From the ten repetitions per stimulus time-resolved (milliseconds) mean firing rates were calculated by means of kernel convolution [50,51] for each single unit. We used an asymmetric kernel with the kernel shape ‘ALP’ (alpha function) and time resolution, *τ*. We applied a baseline correction by calculating the mean activity of 1000 ms before stimulus onset and subtracted that baseline from the units’ firing rate. The resulting mean rates of all units were used to construct stimulus dependent population vectors in the following way. For a given stimulus, *a*, and an ensemble of *n* neurons we constructed the *n*-dimensional rate vector *v* ^*a*^ at each point in time. To calculate the population mean activity the rate vectors were averaged. All vectors together were used for principal component analysis (PCA). The factor loadings or weights of each single unit were used afterwards to order the units with respect to their contribution to the first principal component.

Time-resolved Euclidean distance (*L* ^*2*^-Norm) were calculated for each pair of rate vectors (*v* ^*a*^- *v* ^*b*^) using the following equation *d(t) = (Σ(v*_*i*_^*a*^ *(t)–v*_*i*_^*b*^ *(t))* ^*2*^ *)* ^*1/2*^.

### Statistics

We used the Statistical Toolbox of MatLab. To test for statistical relevance within the whole ensemble of 100 AL-units we compared the means of the stimulus dependent maximal rate distributions using a balanced one-way ANOVA. The factor loadings to PC1 >0.05 were used to extract 32 units which mostly contributed to the odor induced signal variation. We tested the evolution of the maximal mean rate distribution in three successive time windows (50 ms each) using the Friedman-test for dependent data. We tested post-hoc the pair-wise differences (six pairs) using the Wilcoxon rank sum test with an adjusted alpha-level of p<0.0083 (0.05/6) to account for multiple comparisons. To test for statistical differences between all six Euclidean distances a two-sided Wilcoxon rank sum test was performed with an adjusted alpha-level of p<0.0033 (0.05/15).

## Results

**Fig 1:**
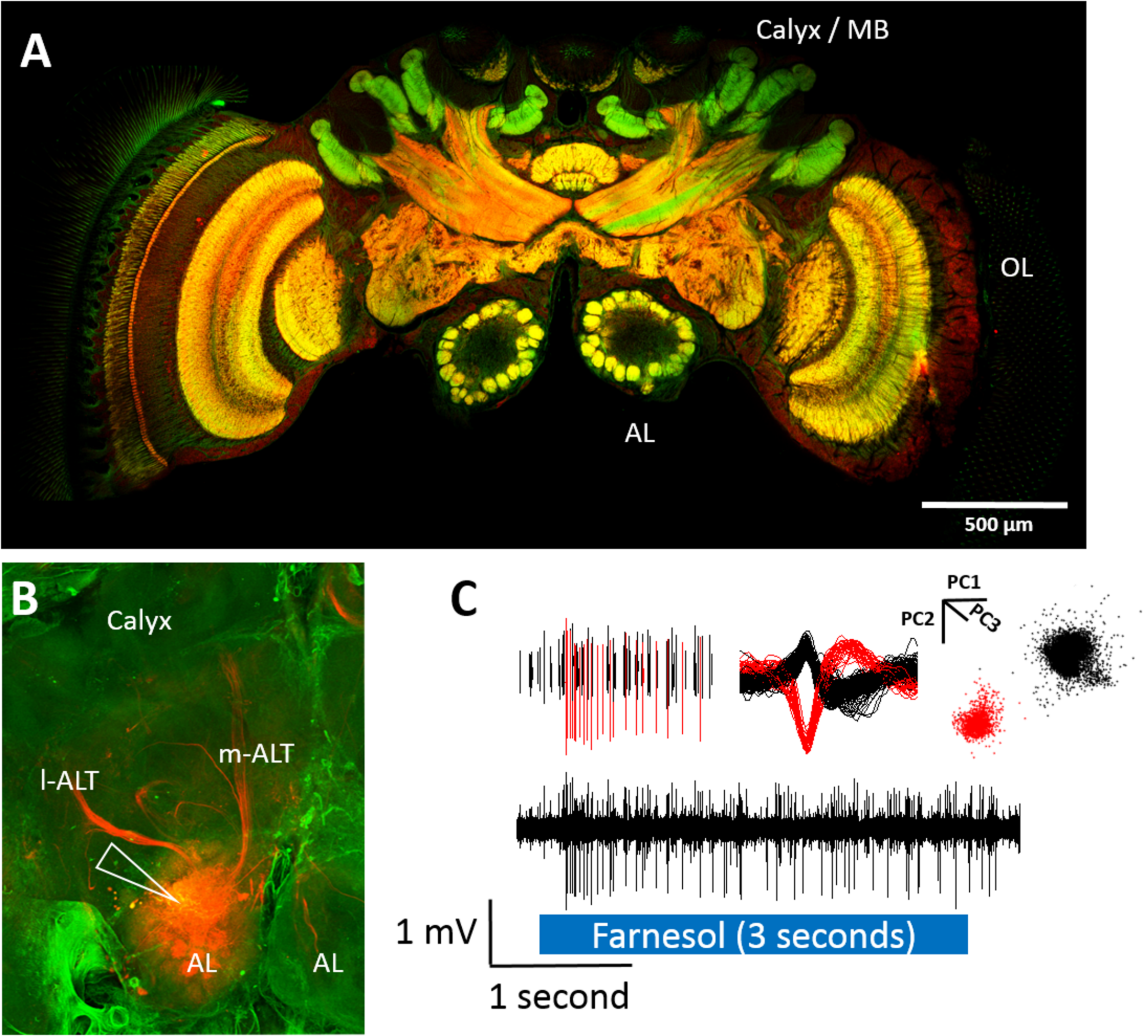
Extracellular recordings of antennal lobe (AL) activity in bumble bees *(Bombus terrestris)*. (A) Overview of the brain of a *Bombus terrestris* worker (AL = antennal lobe, OL = optical lobe, MB = mushroom body), immunostained with anti-synapsin (red) and fluorophore conjugated phalloidin (green). (B) After recording we used a standard dual staining technique resulting in yellow fluorescence tissue around the electrode’s tip (white arrow) to verify the position in the AL. In addition, the lateral (l-) and the medial (m-) antennal lobe tract (ALT) was labelled (for details see Brill et al. (49)). (C) Example of a response to farnesol (blue bar). The signal to noise ratio of the extracellularly recorded activity (black trace) allows a clear separation of single unit activity. The upper panel illustrates the waveform overdraw of two units (black and red) and the unit separation after principal component analysis. The first three principal components (PC1-3) of the whole recording trace (150 seconds) were plotted against each other. Each dot refers to one action potential.

We recorded extracellular neural activity close to the dorsal rim of the AL in worker bumble bees (Fig 1). In contrast to bumble bee drones, workers do not possess morphologically distinct or segregated macro glomeruli (unpublished data). We obtained a relatively high signal to noise ratio including well pronounced action potential waveforms which we used to extract single unit activity (Fig 1C). The units we recorded showed diverse response combinations to the different odorants.

### Different odor response profiles despite a balanced rate distribution

**Fig 2:**
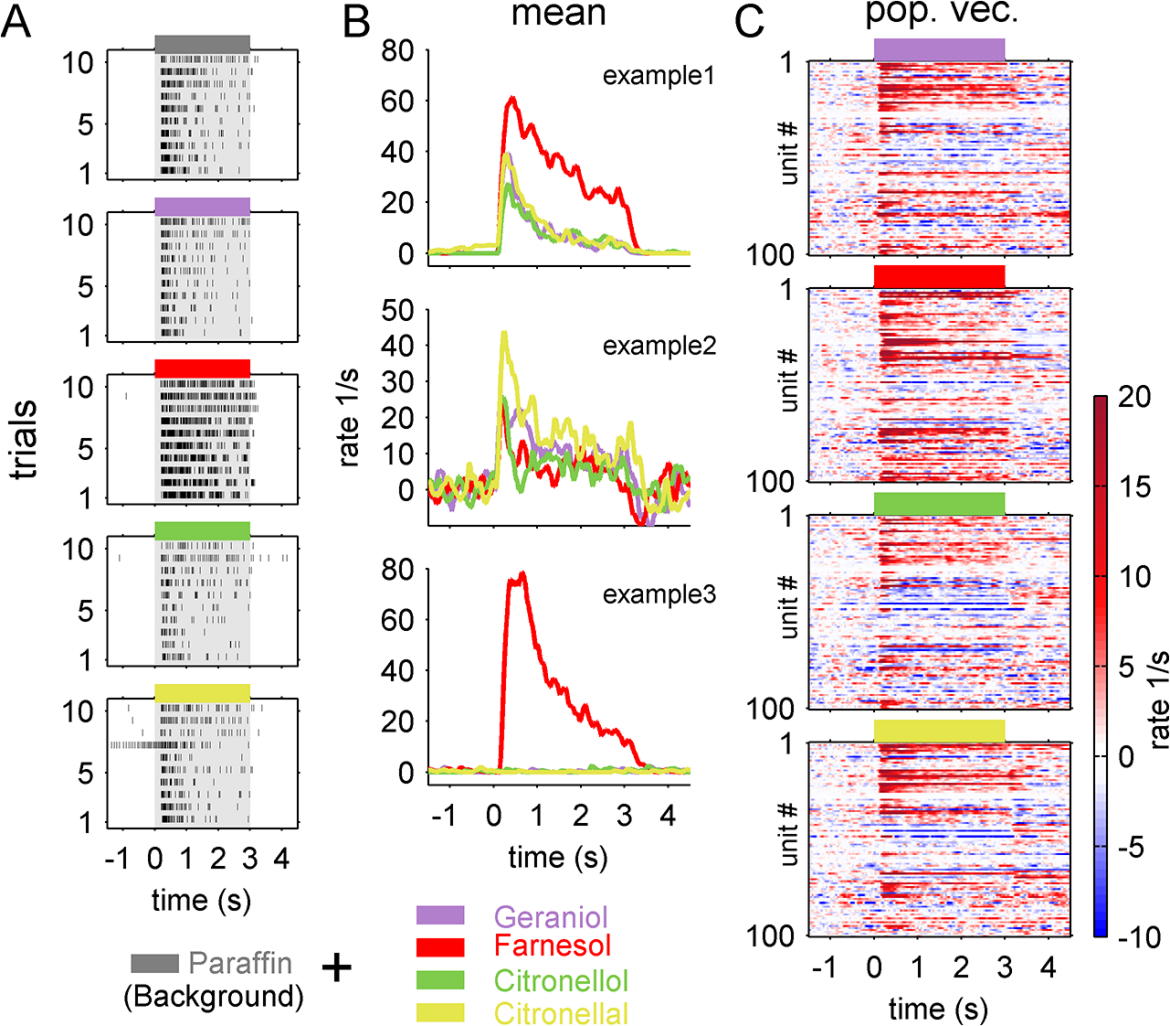
Experimental procedure and construction of population vector activity. (A) (example unit); trial resolved single unit activity in response to the different odor stimuli. Each tick mark refers to one action potential (spike). The colored bars on top of each spike raster plot indicates the three seconds of odor presentation (grey = Paraffin oil, used as solvent and background, violet = Geraniol, red = Farnesol, green = Citronellol, yellow = Citronellal; cp. legend below). The example unit is responding reliably to each single stimulation (trial). From the 10 trials we calculated the odor dependent average response rates for each single unit. (B) Averaged response rates of three example units (color code is given in the legend). Example1 is the unit shown in (A) responding to all of the presented odor stimuli, showing the highest and most prolonged amplitude to farnesol. Example2 also responded to all odors, but the highest response was evoked by citronellal. Example3 did not respond to any other tested odor, but to farnesol. (C) from the averaged response rate of all 100 AL-units the population vectors were constructed. Each single line refers to the averaged response rate out of the 10 trials for a single unit. Odor stimulation started at time zero and lasted three seconds. Response strength is indicated by the color bar.

During the experimental procedure each of the different odorants and the background (paraffin oil) was presented ten times in pseudorandomized order. Fig 2A shows a trial resolved spike raster plot of the single action potentials of an example AL-unit in response to the background and the four tested odor components. If a neuron responded to an odor it usually evoked reliable responses in each single trial. Out of the ten trials of each stimulus we calculated the mean response of every unit for the different stimuli. The recorded units responded with different odor response profiles. Three examples are shown in Fig 2B. Example 1 (upper panel) is the unit shown in Fig 2A responding to all stimuli but being mainly excited by farnesol. Example unit 2 (middle panel) also responded to all odors, but the strongest response was evoked by citronellal. Example unit 3 (bottom) was tuned to farnesol and no other tested odor component evoked a response. The odor dependent mean firing rates of all 100 AL-units were used to construct population rate vectors (Fig 2C). All population vectors revealed a typical temporal diversity of single AL-neuron responses. Some neurons showed phasic, some had tonic responses. Other units showed combinations of both, did not respond at all, or were inhibited. However, comparing the odor induced maximal rate distributions from the 100 AL-neurons, revealed no statistical differences (ANOVA; p = 0.12; f = 1.85, data not shown). We therefore included the different neural firing behaviors of all units and analyzed their ensemble activity. Two types of population response analyses were performed. First, we applied principal component analysis to extract and analyze the units dominating the odor induced activity. Second, we calculated Euclidean distances between the pair-wise population vectors and analyzed the population response differences between the odor representations.

### Typical AL-population response dynamics in *Bombus terrestris*

**Fig 3:**
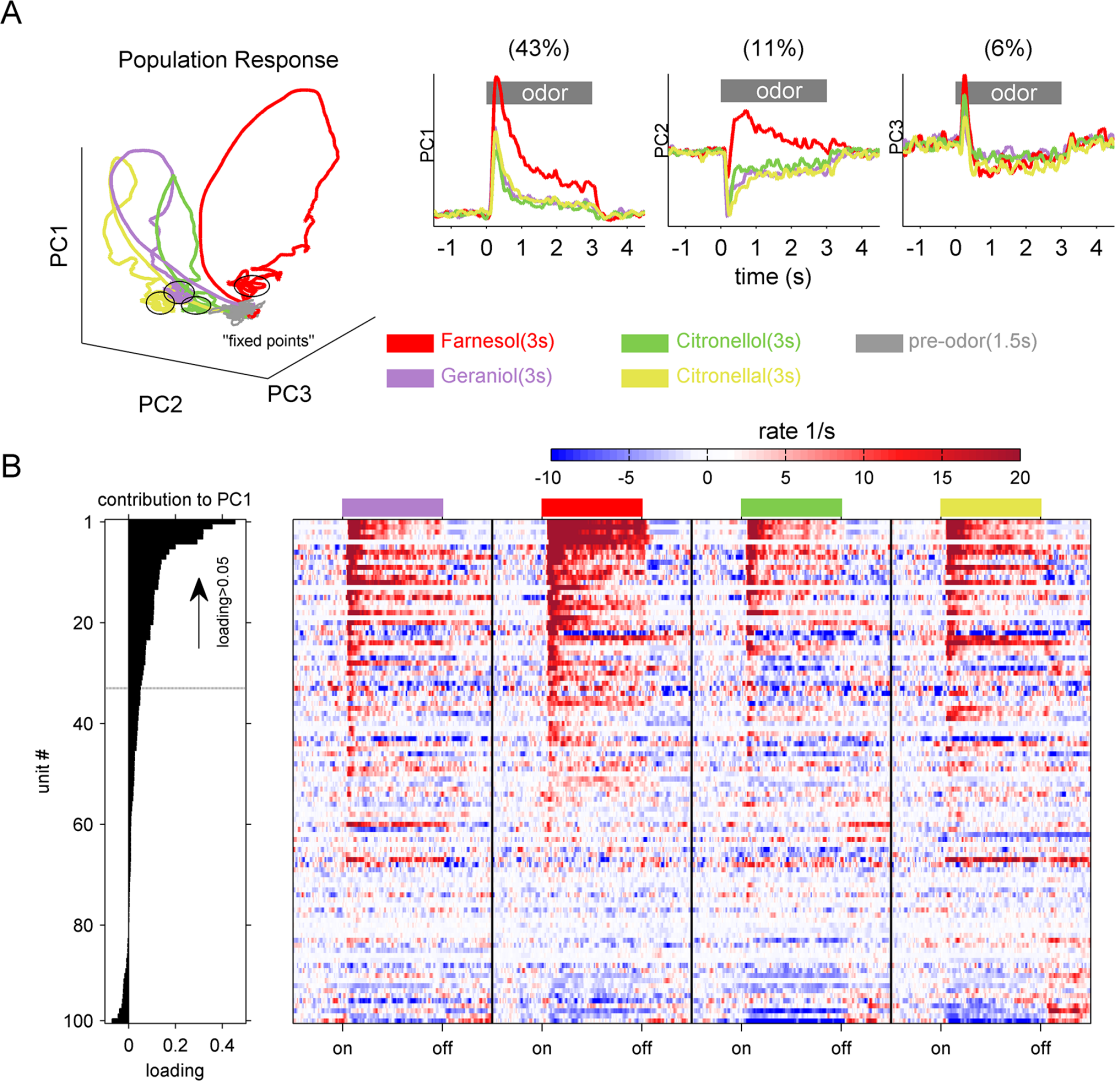
Principal component analysis revealed a prominent combinatorial code extraction of farnesol. (A) Trajectories of the first three principal components (PC1-3) indicate a clear odor separation by the population activity of the 100 AL-units. PC1, PC2 and PC3 explained 43%, 11% and 6% of the variation, respectively. The spontaneous activity before odor onset is shown in grey (Pre-stim). The three second of stimulation are plotted in an odor dependent color code (cp. caption). Note that the population activity for all odors settled in a ‘fixed point’ at around one second after odor onset (cp. PC1-3 time resolved; right three panels), which is separated from the spontaneous activity. The population activity in response to farnesol shows the most separated trajectory, which is also illustrated separately for each of the first three PCs on the right. (B) The factor loadings (left panel) were used to rank the recorded single units with respect to their contribution to the variation in PC1 starting with the most contributing units at the top. The color coded mean firing rate of the single units illustrates that PC1 contrasted units being excited (positive loadings) by the odor stimuli from a few units being inhibited (negative loadings). Furthermore, the colored matrix suggests a spatial code, e.g. unit 5, 14 and 20 were responding only to farnesol and none of the other stimuli. To further analyze odor induced activity, we set a threshold and extracted all units showing factor loadings >0.05.

To be able to analyze the 100 AL-units as a neural ensemble or population including the whole spectrum of neural response patterns (phasic-, tonic-, excitatory- and inhibitory-responses), we used principal component analysis (PCA). PCA was performed taking into account time as the source of sample points, and number of neurons as the dimensions of the original component space. Comparison of the time courses of the ensemble responses of the first three principal components (PC1-3) revealed a clear separation of all stimuli by the AL-unit population (Fig 3A). Prominent transients were formed in response to all odors during the first second after odor onset, settling into a ‘fixed point’ dynamic lasting for the remaining seconds of odor stimulation. The latter was described to occur in AL activities of different insect species [47,52]. The time courses of the first three principal components (Fig 3A right) illustrate a unique separation of farnesol from the other tested odorant stimuli. PC1 explained 46% of variation in the AL-ensemble, showing a clear farnesol induced activity lasting for the three seconds of odor stimulation. The other stimuli induced comparable activity. PC2 explained 11% of the firing rate variation. After following the same direction of activity during the first few hundred milliseconds, it positions farnesol in opposite direction relative to the other odors during a later phase of odor presentation. PC3 explained 6% of the variation describing similar odor induced activity for all stimuli tested.

### Delayed and prolonged separation of farnesol is accompanied by recruitment of specialized single AL-units

**Fig 4:**
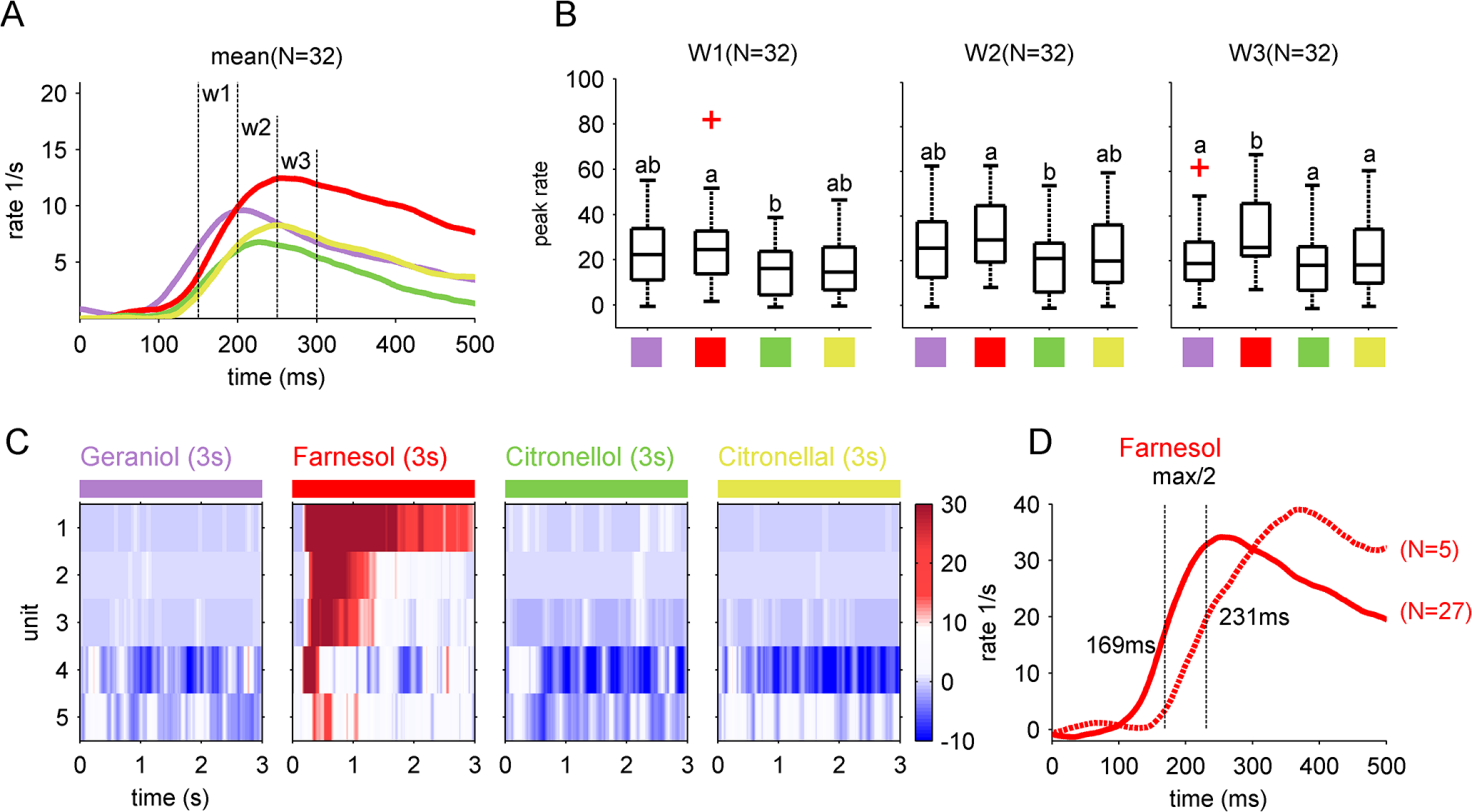
Separation of farnesol increased over time. (A) Odor dependent mean rates of the 32 units showing factor loadings >0.05 (Fig 3) during the first 500 ms of odor stimulation (time 0 marked the odor onset). Three time windows (w1-w3; 50 ms each) were used to compare the odor induced activity. (B) Maximal mean rate distribution for the three time windows. The central mark in each box indicates the median, the edges of the boxes were the 25th and 75th percentiles, and the whiskers extend to the most extreme data points. Outliers are marked in red. A Friedman test revealed significant differences in all three time windows (p<0.05). The pairwise differences in each single window were tested using a Wilcoxon rank sum test. Same letters indicate statistical similarity (p>0.0083). In window 1 and 2 the maximal mean rate distribution of farnesol and citronellol were significantly different. In the last window (W3), 250–300 ms after odor onset, only farnesol evoked a significantly increased activity compared to the other tested odors. (C) Color coded mean rates of units which responded to farnesol only. (D) The mean rate of the 5 units (shown in C) were compared with farnesol induced mean rates of the other 27 units that showed loadings >0.05 (Fig 3). Both rates were shifted in time. The units responding to farnesol only reached their half maximal mean rate 231 ms after odor onset, which was ~62 ms delayed compared to the mean rate response of units responding to farnesol and other odors.

Interestingly, farnesol was separated most clearly from all other stimuli. To visualize which neurons were responsible for that separation, we extracted AL-neurons dominating the PCA. We used the factor loadings to rank the recorded single units with respect to their contribution to the variation in PC1 (loading) and color coded their individual response profiles over time (Fig 3B). PC1 contrasts AL-units which were excited by the odors (positive loadings) from units being inhibited (negative loadings). Units showing no odor induced activity were situated in between. In addition, the colored matrix illustrates that a spatio-temporal code seemed to be responsible for the prominent separation of farnesol in PC-space. For example, some units (5, 14 and 20) responded only to farnesol, but to none of the other stimuli. Other AL-units showed higher and temporally distinct responses to geraniol and citronellal, whereas the other stimuli including farnesol evoked less clear rate increases (Fig 3B). We extracted 32 units which showed factor loadings >0.05 and analyzed them separately. Their averaged mean firing rates suggest that a delayed temporal evolution of the AL-signal was responsible for separating the ensemble responses to the different odor stimuli (Fig 4A). In an early phase, ~150–200 ms after odor onset (window1 = w1), the mean rate of geraniol was the highest. During the following 50 ms (200–250 ms after odor onset, w2) the mean rate of farnesol and citronellal increased further, whereas the geraniol and the citronellol mean rate already decreased. During the third test-window (w3, 250–300 ms after odor onset) the mean rate activity of farnesol was clearly separated from all other stimuli. To test if this temporal effect was of statistical relevance, we compared the maximal rate distributions of the 32 extracted units in the different time windows (Fig 4B). During the early response phase (W1) maximal rate distribution of farnesol was significantly increased compared to citronellol (Friedman test, p<0.05, followed by a Wilcoxon rank sum test, p<0.0083). The same effect was present in the second test window (W2). Starting with the third window (W3) the farnesol maximal rate distribution was significantly increased compared to all other tested odors (Friedman test, p<0.05, followed by a Wilcoxon rank sum test, p<0.0083). We observed five units (recorded in 3 different animals) including unit (5, 14 and 20) shown in Fig 3B which responded to farnesol only (Fig 4C). All other units responded to more than one stimulus. We compared the farnesol induced averaged response rates of these 5 units to the averaged response rates of the other 27 of the 32 selected units (loadings >0.05). Interestingly, we found a run time difference of ~62 ms to reach half maximal mean rate response (Fig 4D). This indicates that the late responding 5 farnesol tuned units were likely to be recruited by the network to reach maximal mean rate responses between 300–400 ms, which was after the third time window we tested.

### Prolonged computation of farnesol in the AL-ensemble results in a higher separation

**Fig 5:**
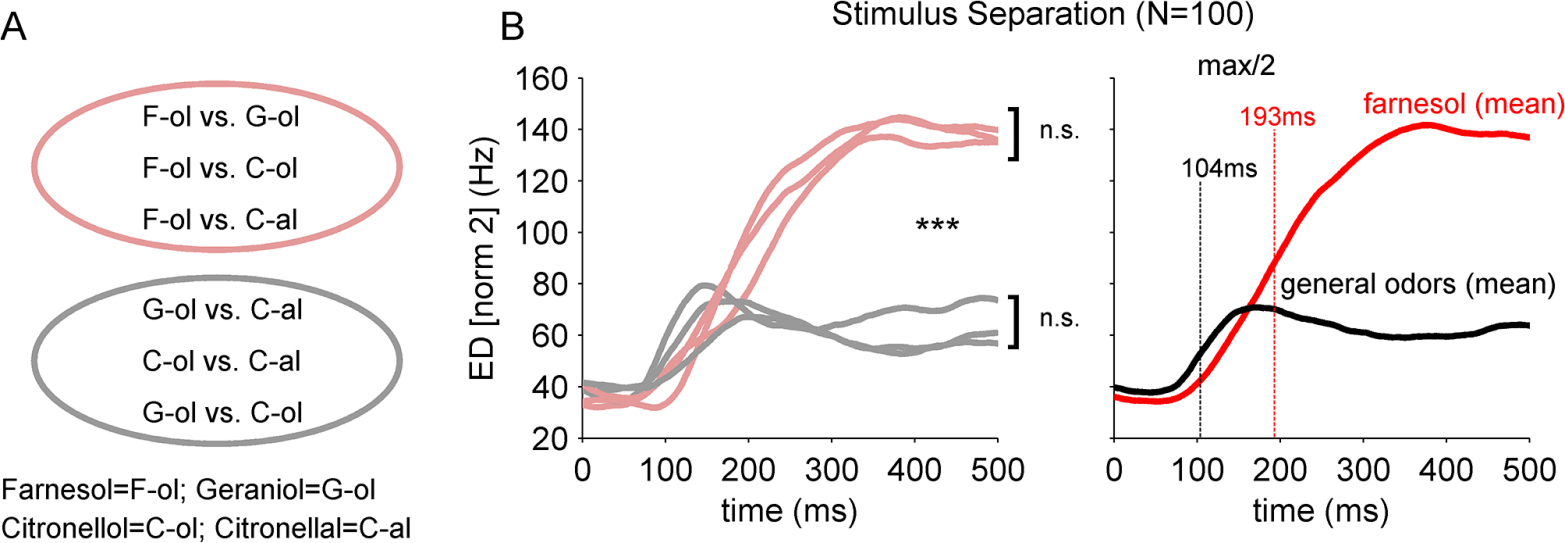
Prolonged and increased separation of farnesol in the AL-network. (A) Out of the population vectors of the four different odor representations six pairwise Euclidean distances of second norm (ED [norm2]) were calculated. Three EDs illustrate the separation of farnesol from other tested odors (pink) and three ED pairs include the separation among the other (non-farnesol) odor stimuli (grey). (B) left; EDs including farnesol showed a clear increase compared to the non-farnesol odor separation. Note, that the EDs including the non-farnesol odors (grey) reached the same amount of odor separation, although two pairs differ in their functional group (alcohols vs. aldehydes; Wilcoxon rank sum test, p<0.003). The EDs illustrating farnesol separation (pink) were not significantly different among each other, but all were significantly different from the non-farnesol pairs (Wilcoxon rank sum test, p<0.003). (B) right; The mean ED out of the population vector pairs including farnesol (red) reached half maximal odor separation ~193 ms after odor onset (time zero), which was prolonged by ~89 ms compared to the mean separation between the non-farnesol (general-) odors (black).

The analysis of the subset of 32 AL-units supports that the farnesol induced activity evolves over time. In the next step we tested if this delayed temporal evolution was also reflected if we treated the 100 recorded AL-neurons as one ensemble. We calculated the Euclidean distances (ED; (norm 2)) between the pairwise population vectors of the different odor stimuli (Fig 5). Using EDs we take into account all occurring stimulus dependent rate contrasts of the single AL-units over time. Fig 5 shows the EDs including the farnesol population vector and the EDs calculated between the population vector couples of the other stimuli (Fig 5A). Compared to the separation of geraniol vs. citronellol, geraniol vs. citronellal and citronellol vs. citronellal (general odors) the separation of farnesol vs. geraniol, farnesol vs. citronellol and farnesol vs. citronellal was increased more than twice (Fig 5B left). Both groups differed significantly (Wilcoxon rank sum test, p<0.003). Furthermore, separation maxima for the later pairs occurred 300–400 ms after odor onset, whereas the general odor pair distances reached maximal separation between 100–200 ms after odor onset, which was more than 100 ms earlier. In addition, the separation of farnesol from another alcohol (same functional group) or farnesol from an aldehyde (different functional group) did not differ (Wilcoxon rank sum test, p>0.003, n.s. after correction for multiple comparisons). Also, the separation between the general odors did not differ depending on the functional group (Wilcoxon rank sum test, p>0.003). On average, the AL-network needed 104 ms to reach half maximal odor separation for the tested general odors, whereas reaching half maximum separation of farnesol took an additional 93 ms (Fig 5B right).

## Discussion

Our results show that farnesol evoked a distinct and prolonged population activity of the AL-neuron network ensemble which was significantly increased compared to all other tested chemically related odor molecules. This most likely reflects a unique computation of farnesol within the AL-network that may enhance extraction of the biological relevance of this pheromone component in bumblebee workers.

### Typical single-unit and population activity in bumblebee AL-neurons

In honeybees, AL neurons typically respond with very fast high-frequency on-responses, constant tonic firing patterns, combinations of both, or inhibition. These neural firing behaviors are characteristic for both, PNs [35,37,53,54] and LNs [35,54,55] documented by intracellular recordings combined with morphological staining. Similar patterns have been observed in *Drosophila melanogaster* [55–57], *Bombyx mori* [58], *Agrotis ipsilon* [14], and *Schistocerca americana* [52,59]. Furthermore, these response dynamics have also been reported for mitral cells in the olfactory bulb of the zebrafish *Danio rerio* [60,61]. In the bumblebee *Bombus terrestris* we also found these characteristic firing patterns of AL-neurons (Figs 2 and 3) that resulted in a typical AL-population activity as visualized by principal component analyses. The fast ‘transient’ change from baseline activity, followed by a so called ‘fixed point’ activity one second after stimulus onset, which persisted for the duration of the stimulus application (Fig 3), seems to be characteristic for insects in general [47,52].

### Prolonged spatio-temporal segregation separates farnesol from other stimuli

The neural activity of the AL consists of a complex network of temporal excitation and inhibition, resulting in a spatio-temporal pattern coding for an odor stimulus [33–35]. The spatial segregation of this pattern starts already at the AL-input level, where OSNs expressing the same OR converge on spatially distinct glomeruli [62–66]. At the AL-output level (the level of our recordings) it has been suggested that in honeybees the glomerular pattern becomes sharpened to increase the contrast between different odor stimuli [33], whereas in *Drosophila* a broader odor tuning [67] or an increase in the degree of odor separation by contrast enhancement [34,68] and odor dependent modulation could be observed [69]. Both types of spatio-temporal modulations may explain the distinct separation of farnesol at the AL output level we observed as both result in a change of the proportion of excited and inhibited glomeruli depending on the processed stimulus. When we sorted recorded AL-units according to their contributions to variations in PC1 (Fig 3B), we observed that units showing a high activity in response to farnesol were less activated during stimulation with e.g. geraniol and vice versa. In the extreme, we found 5 units that responded only to farnesol, but not to the other tested stimuli. This kind of unit-specialization was not found for any other tested odor. Moreover, the response of the farnesol-tuned units was delayed by about 62 ms. This delayed activation might be the result of a prolonged computation in the local AL-network. The farnesol separation by the ensemble of 100 AL neurons was increased and prolonged by ~89 ms compared with the separation of the general odors (Fig 5). A similar phenomenon was described to occur at the mushroom body output level after associating an odor with a reward stimulus. Here, some initially silent units became recruited during memory retention to compute for the rewarded odor [23]. As a consequence, the population activity for the reward associated stimulus (CS+) was prolonged resulting in a delayed stimulus separation for the behaviorally relevant stimulus (CS+). We thus assume that a spatio-temporal odor dependent activity distribution in the AL network was responsible for the clear prolonged farnesol separation. The underlying mechanism may include hardwired components representing a ‘network-pre-tuning’ for farnesol at the AL-network level. For example, in *Drosophila melanogaster*, only a single receptor type (Or83c) in OSNs innervating only one glomerulus (DC3) facilitates the farnesol response [70]. However, our recordings indicate a combination of both single glomerulus tuning, as illustrated by the units responding to farnesol only, and population network tuning, as the ensemble comprises a variety of neural response behaviors (Figs 2B, 2C and 3B). The combination of both provides a prolonged but reliable separation of farnesol information from the responses of other odors. We therefore suggest a pre-tuning of the AL network, which allows to segregate and extract the behaviorally relevant stimulus already at this first-order processing level in order to induce a fast and adequate innate behavioral response in downstream networks.

Bumblebees can be easily trained to differentiate odors under controlled laboratory conditions [21]. Combined with the established electrophysiological access we are now able to test whether the specific tuning of the AL network to farnesol is fixed or can be modified and ‘rewired’ after linking for example farnesol with a new valence.

### Separation of the behaviorally relevant stimulus by the AL-network

To investigate insect-plant or insect-insect interactions it is important to test how biologically relevant stimuli are detected and processed. A common way is to record antennal activity (electro-antennogram; EAG) during odorant stimulation [43,71–73]. Here we moved one step further along the olfactory pathway and recorded activity at the output level of the AL, the first order olfactory neuropil. At this processing level the innate valence of an odor is represented in a spatial activation pattern of glomeruli, telling the insect to avoid or to approach a specific odor [74]. As mentioned above, in the extreme the activity of a single glomerulus (DC3) can be sufficient to facilitate attraction, like in the case of ripe citrus fruits in *Drosophila* [70]. As the DC3 glomerulus is one of the smaller glomeruli and has less than one third of the volume compared to the larger glomeruli [75], it is innervated by fewer OSNs [76], and accordingly its contribution to, for example an average rate activity, might be hardly noticeable. However, its activation is of enormous relevance for the animal to be able to extract the behaviorally relevant stimulus. Using AL- population response analysis we were able to show a distinct representation of farnesol, a component of the recruitment pheromone in bumblebees, from all other tested odors. Farnesol as a sesquiterpenoid has a molecular weight of 222, whereas geraniol (154), citronellol (156) and citronellal (154) as monoterpenoids are much lighter. The vapor pressure of farnesol (3.9 * 10^5^ mm Hg) is 1000 times lower compared to the other odor compounds tested (geraniol: 3.0 * 10^2^ mm Hg; citronellol: 2.0 * 10^2^ mm Hg; citronellal: 2.8 * 10^2^ mm Hg). The odor concentration of farnesol in the headspace was therefore much lower compared with the monoterpenoids. The concentration of odor molecules in the headspace of the antenna usually correlates with the number of activated OSNs, and thus farnesol activation should be lower compared to the activation by geraniol, citronellol and citronellal. However, this was not the case, supporting our hypothesis of a distinct neuronal representation of farnesol in the AL-network.

The prominent perception of farnesol might be conserved in different insect species since farnesol can be found as a compound in floral volatiles in many plant families [46,77], and as intracellular metabolite it is part of the isoprenoid metabolism [78]. It might, therefore, be a good candidate to investigate functional diversification in plant-insect interactions via chemical cues, and the evolution of pollinator-flower relationships. Our results strongly suggest that the AL-network as a whole may comprise innate representations suitable to separate behaviorally relevant stimuli.

## Conclusion

Here we report, for the first time, multi-unit extracellular recordings and analyses of individual AL-neuron activities in bumblebees. Using population response analyses we show that farnesol, a component of the recruitment pheromone in bumblebees inducing an innate behavioral response, evokes a unique activity pattern at the AL network level compared to other odor stimuli used for localizing floral resources. To confirm, if this effect is a general feature of the *Bombus terrestris* AL or if it is related to its pheromonal nature, we plan additional experiments comparing different bumble bee casts, in particular workers and drones. Our results further show that the electrophysiological population analyses are a useful tool to investigate the perceptual space of behaviorally relevant odor stimuli in insects.
