# Correction: Extracting the Behaviorally Relevant Stimulus: Unique Neural Representation of Farnesol, a Component of the Recruitment Pheromone of *Bombus terrestris*

**DOI:** 10.1371/journal.pone.0139296

**Published:** 2015-09-25

**Authors:** Martin F. Strube-Bloss, Austin Brown, Johannes Spaethe, Thomas Schmitt, Wolfgang Rössler

The affiliation for the fourth author is incorrect. Thomas Schmitt is not affiliated with #1 but with #3 Department of Animal Ecology & Tropical Biology, Theodor-Boveri-Institute of Bioscience, Biocenter University of Würzburg, Am Hubland, 97074 Würzburg, Germany.
